# Starch Allowance and Muscle Enzyme Activity in Healthy Standardbred Trotters Trained by Professional Trainers

**DOI:** 10.1111/jpn.14127

**Published:** 2025-05-06

**Authors:** Malin Connysson, Anna Jansson

**Affiliations:** ^1^ Department of Animal Biosciences Swedish University of Agricultural Sciences Uppsala Sweden

**Keywords:** aspartate aminotransferase, creatine kinase, exercise, horse, nutrition

## Abstract

It is generally accepted that plasma muscle enzyme activity of creatine kinase (CK) and aspartate aminotransferase (AST) may increase in racehorses after exercise and racing, indicating muscle fibre damage and/or increased leakage from muscle fibres. However, other studies suggest that starch intake might influence plasma muscle enzyme activity reported postexercise. This study aimed to evaluate the effect of different starch allowances on plasma muscle enzyme activity in Standardbred trotters in professional training. Seventy‐six horses from five professional trainers were sampled pre‐ and postexercise. The trainers had different feeding strategies and fed various amounts of starch to their horses. Postexercise plasma AST activity was higher (*p* < 0.007) for the high (H) and medium (M) starch allowances (451–967 and 988–1429 g/day, respectively) than for horses with low starch allowances (L) (0–268 g/day) (H:8.1 ukat/L (SE 0.5); M: 8.4 ukat/L (SE 0.5); L: 5.8 ukat/L (SE 0.7) (*p* < 0.007)). Postexercise plasma CK activity was higher (*p* < 0.01) for the medium starch allowance group than for the high and low starch allowance groups (H:4.6 ukat/L (SE 0.3); M: 5.9 ukat/L (SE 0.4); L: 3.9 ukat/L (SE 0.4)). In conclusion, this study showed higher plasma muscle enzyme activity of AST and some elevations in CK activity in horses fed high‐starch allowances compared to horses fed low allowances or no starch. In addition, muscle enzyme activity increased in response to the duration of high‐intensity exercise. Management systems aiming for low levels of plasma muscle enzyme activity could accordingly offer diets with low starch (< 450 g/day) contents and perhaps training regimes with shorter durations of high‐intensity exercise. However, the mechanisms behind and the elevations' biological importance, need further investigation.

## 1 Introduction

The scientific literature shows that muscle enzyme activity of creatine kinase (CK) and aspartate aminotransferase (AST) levels in plasma may increase in Standardbreds and Thoroughbreds after exercise and racing (Pösö et al. 1983; Lindholm 1987; Siciliano et al. 1995; Kristensen et al. 2014), indicating muscle fibre damage and/or increased leakage from muscle fibres. CK is an enzyme found in cytosol and mitochondria in tissues where energy demands are high, for example, muscle tissue (Baird et al. 2012). AST catalyses an energy‐providing process that occurs between the mitochondria and cytosol. This enzyme is mainly localized in the skeletal and myocardial muscle, liver and erythrocytes.

The leakage of muscle enzymes can indicate delayed onset muscle soreness (DOMS), muscle injury or muscle diseases such as recurrent exertional rhabdomyolysis (Hodgson et al. 2014). However, in one recent study on healthy Standardbred trotters fed a forage‐only diet, muscle enzyme activity levels were very low after race‐like exercise tests (Connysson et al. 2021). Interestingly, lowered muscle enzyme responses after exercise have also been observed in horses with recurrent exertional rhabdomyolysis (MacLeay et al. 1999a; McKenzie et al. 2003), as well as in healthy athletic horses (MacLeay et al. 1999a; Connysson and Jansson 2022) when fed low‐starch diets. Starch‐rich diets have also been shown to increase the risk of recurrent exertional rhabdomylosis (MacLeay et al. 1999b). This indicates that diet might influence the elevated levels reported postexercise. Starch‐rich diets have also been associated with other health problems such as colic (Gillen and Catherine Archer 2023), gastric ulcers (Vokes et al. 2023) and stereotypic behaviours (Seabra et al. 2021). In addition, feeding limited forage rations to racehorses has been identified as a welfare concern due to the “Five Domains model” (Stallones et al. 2023). A recent study also suggest higher oxidative stress in slaughter horses fed a high‐starch diet compared to a high‐fibre diets (Raspa et al. 2022).

It has been demonstrated that low‐starch versus high‐starch diets alters the metabolism and availability of energy substrates in Standardbred trotters (Jansson and Lindberg 2012; Connysson et al. 2017). A forage‐only diet (low‐starch) increases plasma acetate concentrations and gives lower plasma insulin responses than a high‐starch diet (Jansson and Lindberg 2012; Connysson et al. 2017). In addition, low‐starch diets give lower plasma lactate concentrations during submaximal exercise (KARLSSON et al. 2002; Jansson and Lindberg 2012).

This study aimed to evaluate plasma muscle enzyme activity in Standardbred trotters in professional training offered different amounts of starch. The hypothesis was that lower starch intakes give lowered muscle enzyme activity postexercise.

## 2 Materials and Methods

The Umeå Regional Ethics Committee approved the study, which was performed in compliance with European Union directives on animal experiments (2010/63/EU; European Union 2010), and laws (Swedish Constitution 1988) and regulations (Swedish Constitution 2012) governing experiments on live animals in Sweden.

### 2.1 Horses and Management

This study was carried out from September 2022 to January 2023. Seventy‐six horses from five professional trainers (licensed by the Swedish Trotting Association) were included (7–37 horses at each trainer). The trainers had different feeding strategies and fed various amounts of concentrates to their horses. To be included in the study, horses had to be trained by the trainer for at least 1 month before sampling. Information on sex and age was collected from the Swedish Harness Racing Association. There were 12 colts (C), 28 fillies (F) and 36 geldings (G) and the horses were 2–12 years old. They were trained for harness racing and most of the horses had raced before measurements (*n* = 46) and almost all raced in the years after (*n* = 72). Main earnings in the study population before measurements were 11,790 EUR (range 0–169,805 EUR) and main earnings in the study population in October 2024 were 22,008 EUR (range 0–189,399 EUR).

### 2.2 Sampling and Exercise

Horses were sampled during exercise on two different occasions 1 month apart. The exercise sampled was a part of the horse's normal training programme and was conducted as interval exercise, uphill interval exercise, heat exercise, or competition race. All horses were equipped with a heart rate recorder (Polar M460 Polar Electro, Kempele, Finland) during the exercise on the sampling days. Blood samples were collected 10–60 min before exercise, at rest, and within 9–25 min after the horses had performed their ordinary intense exercise sessions (minutes after the last heat/interval). The mean time from the last heat/interval to sampling was 15 min (range 7–25; SE 0.5 min).

### 2.3 Diet

Information on feed allowances was collected for each horse. The trainers used different commercially available concentrates, and a sample from each concentrate was collected and analysed for starch content (ISO 15914). The mean starch content of the concentrates was 286 (SE 31.6) g/kg DM, ranging from 153 to 467 g/kg DM (composition of concentrates is given in Supporting Information: S1). The total starch intake was then calculated from feed allowances and starch content. Some horses had different concentrate intakes on different weekdays, and a mean daily starch intake per week was estimated for those horses. The horses were divided into three groups based on daily starch intake (low, medium, and high; see Table 1). One horse was in the medium group on the first occasion and in the high group on the second occasion and is therefore included in both groups. All horses were fed in the morning, and all exercises were conducted before lunch. This resulted in the time between feeding and exercise being between one and 4 h.

**Table 1:** Classification of horses in groups based on starch intake.

Starch intake group	Daily starch intake (g)	Number of horses	Horses per trainer	Age	Sex
Low	0–268	18	Trainer 4 (*n* = 6) Trainer 5 (*n* = 12)	2 years (*n* = 3) 3 years (*n* = 1) 4 years (n = 3) 5–12 years (*n* = 11)	Colts (*n* = 1) Geldings (*n* = 8) Fillies (*n* = 9)
Medium	451–967	26	Trainer 1 (*n* = 7) Trainer 2 (*n* = 10) Trainer 3 (*n* = 8) Trainer 4 (*n* = 1)	2 years (*n* = 6) 3 years (*n* = 8) 4 years (*n* = 7) 5–12 years (*n* = 5)	Colts (*n* = 4) Geldings (*n* = 11) Fillies (*n* = 11)
High	988–1429	33	Trainer 1 (*n* = 2) Trainer 3 (*n* = 31)	2 years (*n* = 16) 3 years (*n* = 6) 4 years (*n* = 5) 5–12 years (*n* = 6)	Colts (*n* = 7) Geldings (*n* = 17) Fillies (*n* = 9)

### 2.4 Measurements and Analyses

Blood samples were collected by venipuncture from the jugular vein in 6‐mL lithium‐heparinized tubes (102 IU, BD, Belliver Industrial Estate, Plymouth, UK). They were kept on ice until centrifuged (10 min, 920 × *g*), after which the plasma was frozen (−20°C).

Plasma acetate concentration, glucose concentration, AST activity, and CK activity were analysed in all samples. Plasma activity of AST, CK, and concentrations of glucose were analysed using the Beckman Coulter DxC 700 AU automatic biochemistry analyser with reagents from Beckman Coulter (USA) (CV; AST: 0.56%, CK: 0.93%). Plasma acetate and lactate concentrations were analysed with an enzymatic and spectrophotometric method (Boehringer Mannheim/R‐Biopharm, Darmstadt, Germany). Plasma lactate concentrations were only analysed in the sample taken after exercise using 2500 YSI (CV:2.3%). Plasma insulin concentration was only analysed in samples taken at rest using ELISA 2 (Mercodia equine insulin kit, Mercodia, Uppsala, Sweden) (CV: 2.7%).

Heart rate data were analysed using the software application Polar Flow (Polar Electro, Kempele, Finland).

### 2.5 Statistical Analysis

Statistical analysis of CK, AST, glucose, and acetate was performed in the MIXED procedure in SAS (version 9.4; SAS Institute Inc, Cary, NC, USA) with a model including fixed effects of age class, sex, starch intake group, and the interaction between starch intake group and sample. A MIXED model was used since there were repeated measurements in the horses. The horses were divided into three age classes (class 1: 2 years old; class 2: 3–4 years old; class 3: 5–12 years old). The model for an observed variable of horse (trainer) *i* (experimental unit), age class *j*, sex *k*, starch intake group *l*, sample (at rest and after exercise) *m* was:

$$Y_{\text{ijklm}}=\mu +\eta_{i}+\pi_{j}+\gamma_{k}+t_{l}+\chi_{m}+{\left(t\chi \right)}_{\text{lm}}+e_{\text{ijklm},}$$

where *μ* is the overall mean, *η_i_* is the effect of horse, *π_j_* is the effect of age class, *γ_k_* is the effect of sex, *t_l_* is the effect of starch intake group, *χ_m_* is the effect of sample, (*tχ*)_lm_ is the effect of the interaction between starch intake group and sample and *e*_ijklm_ is the random error. The random part included horse (trainer) and trainer. Observations within each horse were modelled as repeated measurements.

To analyse the effect of the duration of intense exercise on CK, AST, glucose, and acetate, a similar statistical analysis was performed with the exercise duration group included as a fixed effect. For this statistical analysis, exercise sessions were divided into three groups depending on the length of time heart rates > 200 bmp were observed (group 1: 0–180 s; group 2: 198–388 s; group 3: 415–722 s). In this analysis, only horses with accurate heart rate registrations were included (*n* = 68).

For insulin, a statistical analysis was performed in the MIXED procedure, including fixed effects of age class, sex, and starch intake group. The random part included horse. Observations within each horse were modelled as repeated measurements.

Starch allowance was analysed using the GLM procedure in SAS with a model including sex, age class, exercise duration group, and the interaction between sex and age class.

Values are presented as least‐squares means (LSM) with standard error (SE). Differences were considered statistically significant at *p* < 0.05.

For heart rates, duration of heart rates over 200 beats per minute and plasma lactate, the mean values and standard errors were calculated using Excel (version 2016, Microsoft Corporation, Redmond, WA, USA), with values presented as means and standard errors. Duration of heart rates over 200 beats per minute and plasma lactate were analysed using the GLM procedure in SAS with a model including trainer and starch intake group.

## 3 Results

### 3.1 Starch Allowance

Mean daily starch allowances were lower for fillies compared to colts and geldings (fillies 588 g (SE 70); colts 1043 g (SE 123); geldings 832 g (SE 61); (*p* < 0.01)). There was a tendency (*p* = 0.06) for the younger horses (class 1) to have higher starch allowances than the oldest horses (class 3) (class 1: 930 g (SE 81); class 2: 859 g (SE 74); class 3: 675 g (SE 109)). There was no effect of the exercise duration group on mean daily starch allowances (exercise group 1: 735 g (SE 70); exercise group 2: 829 g (SE 77); exercise group 3: 902 g (SE 93)).

### 3.2 Exercise

During exercise, the horses had an average maximal heart rate of 215 (SE 1.3) beats per minute (bmp) ranging from individual max heart rate registrations of 172–237 bmp. The average duration of exercise with a heart rate over 200 bmp was 258 (SE 20) seconds with a range of 0–722 s. The average plasma lactate concentration postexercise was 3.1 (SE 0.6) mmol/L with a range of 0.5–34 mmol/L. There was no significant difference between starch intake groups on plasma lactate concentrations or duration of exercise with heart rates over 200 bpm.

### 3.3 Effect of Starch Allowance and Exercise

Plasma AST activity (ukat/L) was higher after exercise than before exercise on all starch diets (Table 2). Plasma AST activity was significantly lower on low starch intake than on medium and high starch intake both before and after exercise (Table 3). Plasma CK activity (ukat/L) was higher after exercise on medium starch intake compared to low and high starch intake (Table 2).

**Table 2:** Plasma activity of creatine kinase (CK), aspartate aminotransferase (AST), and plasma concentrations of glucose, acetate, and insulin in trained Standardbred trotters offered three different starch allowances. *p*‐values describe differences between samples taken before and after exercise. Values are presented as least‐squares means (LSM) with standard error (SE).

Variable	Starch intake	Before exercise	After exercise	SE	*p*‐value (before vs. after exercise)
Plasma AST	High	7.6^a^	8.1^a^	0.5	< 0.0001
(ukat/L)	Medium	7.9^a^	8.4^a^	0.5	< 0.0001
	Low	5.4^b^	5.8^b^	0.7	< 0.0001
Plasma CK	High	4.2^a^	4.6^a^	0.3	0.203
(ukat/L)	Medium	3.9^ab^	5.9^b^	0.4	< 0.0001
	Low	3.0^b^	3.9^a^	0.4	0.089
Plasma glucose	High	5.7	6.3	0.2	0.003
(mmol/L)	Medium	5.5	6.0	0.2	0.032
	Low	5.1	5.8	0.3	0.009
Plasma acetate	High	0.78^a^	0.67^a^	0.03	0.001
(mmol/L)	Medium	0.66^b^	0.67^a^	0.04	0.862
	Low	0.83^a^	0.89^b^	0.05	0.150
Plasma insulin	High	21.7^a#^	—	2.2	—
(mU/L)	Medium	14.2^b^	—	2.4	—
	Low	15.3^¤^	—	3.0	—

^a,b^Different letters in the same column = significant (*p* < 0.05) difference between starch intake groups. ^#¤^Different symbols in the same column = tendency (*p* < 0.10) for difference between starch intake groups.

**Table 3:** Effect of duration of intense exercise on postexercise plasma activity of creatine kinase (CK), aspartate aminotransferase (AST), and plasma concentrations of glucose and acetate according to the three exercise groups. Values are presented as least‐squares means (LSM) with standard error (SE).

Variable	Exercise group	After exercise	SE
Plasma AST	1	7.2^a^	0.5
(ukat/L)	2	7.7^ab^	0.4
	3	8.2^b^	0.5
Plasma CK	1	4.3	0.6
(ukat/L)	2	4.5	0.6
	3	5.1	0.9
Plasma glucose	1	5.8	0.3
(mmol/L)	2	6.1	0.3
	3	6.3	0.5
Plasma acetate	1	0.74^a^	0.04
(mmol/L)	2	0.78^a^	0.04
	3	0.93^b^	0.05

^a,b^Different letters in the same column = significant (*p* < 0.05) difference between exercise groups.

Plasma glucose concentrations were higher after exercise than before on all starch intakes (Table 3), but there were no differences in glucose concentrations between starch intake groups. Plasma acetate concentrations were lower after exercise than before in horses with high starch intakes. Before exercise, plasma acetate concentrations were higher in horses with high starch intakes and low starch intakes than in horses with medium starch intakes. After exercise, plasma acetate concentrations were higher in horses with low starch intakes than in horses with high and medium starch intakes (Table 2).

Horses on high starch intake had higher plasma insulin concentrations than horses on medium starch intake and a tendency (*p* = 0.09) to have higher plasma insulin concentrations than horses on low starch intake (Table 2).

### 3.4 Effects of Sex

There was a general effect, including all starch intakes and before and after exercise samples, of sex on both plasma AST activity and plasma CK activity. Plasma AST activity ((F:8.6 ukat/L (SE 0.5); C: 6.5 ukat/L (SE 0.8); G: 6.5 ukat/L (SE 0.5) (*p* < 0.04)) and plasma CK activity ((F:5.0 ukat/L (SE 0.3); C: 3.8 ukat/L (SE 0.4); G: 4.0 ukat/L (SE 0.2) (*p* < 0.02)) were higher for fillies than colts and geldings. There was no significant difference between fillies and colts on plasma glucose concentration, but fillies had higher plasma glucose concentration than geldings (*p* = 0.019) ((F: 6.0 mmol/L (SE 0.2); C: 5.7 mmol/L (SE 0.2); G: 5.6 mmol/L (SE 0.1)). There was no significant difference between sexes on plasma acetate concentrations (M: 0.72 mmol/L (SE 0.03); C: 0.79 mmol/L (SE 0.05); G: 0.74 mmol/L (SE 0.03)) or plasma insulin concentrations (F: 15.0 mU/L (SE 2.1); C: 20.1 mU/L (SE 3.5); G: 16.1 mU/L (SE 1.9)).

### 3.5 Effects of Duration of Intense Exercise

Duration of intense exercise only affected plasma AST activity and plasma acetate concentrations. Plasma AST activity was lower in exercise group one than in exercise group three (Table 3). Plasma acetate concentrations were lower in exercise groups one and two than in exercise group three (Table 3).

## 4 Discussion

Traditionally, the high‐energy requirement of athletic horses has been met by feeding large amounts of high‐energy concentrates rich in starch. A review of feeding practices for racehorses in the USA, Germany, Australia and Sweden between 1979 and 2007 reported concentrate allowances of 6.8 ± 0.4 kg/day and forage allowances of 5.8 ± 0.4 kg/day (Jansson and Harris 2013). Even if much starch‐rich concentrates are still fed to racehorses it has been shown that Standardbred trotters, subjected to intense training and competition, can maintain body condition and performance on forage‐only diets with forage containing 10.4‐11.7 MJ ME per kg DM (Connysson et al. 2006; Muhonen et al. 2008; Jansson and Lindberg 2012; Ringmark 2014). Interestingly, it has also been suggested that forage‐only diets can have advantages, compared to forage‐concentrate diets, on performance parameters such as higher lactic acid threshold and mitigated acidosis (Jansson and Lindberg 2012) as well as lower heart rate and plasma cortisol concentrations during transportation (Connysson et al. 2017; Jansson et al. 2018).

The results from this study indicate that both starch intake and high‐intensity exercise affect plasma CK and AST activity levels in healthy trained Standardbred trotters. Increased muscle enzyme activity after intense exercise and racing has been shown in many earlier studies (Pösö et al. 1983; Lindholm 1987; Siciliano et al. 1995; Kristensen et al. 2014). Muscle enzyme activities in the present study were relatively low and could reflect that blood samples were collected relatively quickly (7–25 min) after exercise. CK reaches peak concentrations within a few hours after exercise, and AST reaches peak concentrations after approximately 24 h (Harris et al. 1998). Since this was a field study performed on privately owned horses during normal exercise, the number of blood samples taken had to be restricted and the possibility of controlling sampling time was limited. The limited possibility to control sampling time was also reflected in the low average plasma lactate concentrations, which were probably due to the cool‐down jogging of the horses 7–25 min before blood sampling. The disappearance of blood lactate has been shown to increase if the horse is active during short‐term recovery (Marlin et al. 1987).

Lowered muscle enzyme activity as an effect of low or no starch intake is in accordance with earlier smaller controlled studies in healthy horses (MacLeay et al. 1999a; Connysson and Jansson 2022) and in horses with recurrent exertional rhabdomyolysis (MacLeay et al. 1999a; McKenzie et al. 2003). The lower plasma CK values in the high‐ and medium‐starch intake groups in the present study compared to the concentrate diet group in Connysson and Jansson (2022) (4.6 and 5.9 vs. 12.2 ukat/L) might be explained by higher starch intakes in Connysson and Jansson (2022) than in the present study (1673–3100 g starch/day vs. 451–1429 g starch/day). The elevated AST plasma activity on the high‐starch intake diets in the present study and Connysson and Jansson (2022) could indicate that plasma AST is elevated from the previous exercise session before the sampled one. Most Standardbred trotters have a training programme with 72–96 h between exercise sessions (Ringmark 2014). Plasma AST has a half‐life of 7–10 days (Cardinet et al. 1967).

Since elevated muscle enzyme activity in plasma is an indicator of muscle damage and/or increased leakage, it could be speculated that starch intake affects the level of this leakage. During exercise, the skeletal muscle contractions generate free radicals, and intense exercise can result in oxidative damage to the muscle fibre (Powers and Jackson 2008). A higher risk for oxidative stress in horses fed a high‐starch diet compared to a high‐fibre diet has been suggested in slaughter horses due to lower plasma concentrations of catalase in the horses fed the high‐starch diet (Raspa et al. 2022). Catalase is an antioxidant enzyme that is distributed throughout the cell and occurs more in muscle fibres with high oxidative capacity and less in fibres with low oxidative capacity (Powers and Jackson 2008).

Increased muscle enzyme activity in plasma is also an indicator of more severe muscle damage such as exertional rhabdomyolysis (Valberg 2025). Exertional rhabdomyolysis describes horses with acute clinical episodes of muscle stiffness, cramping, and muscle damage (Valberg 2025). There are, however, many different underlying causes. One of the causes of exertional rhabdomyolysis is polysaccharide storage myopathy (PSSM). In horses with PSSM, diets low in starch have been shown to give lower variation in glucose and insulin concentrations and a more normal serum CK response during exercise compared to diets high in starch intake (Ribeiro et al. 2004). Horses with PSSM have elevated storage of muscle glycogen compared to normal horses (Valberg et al. 2011). Elevated glycogen stores in connection with insulin sensitivity has been suggested to be connected to exercise‐induced rhabdomyolysis in PSSM horses, although the mechanisms are unclear (Ribeiro et al. 2004). Studies on Standardbreds comparing low‐/no‐starch diets with a high‐starch diet show higher muscle glycogen before and after exercise in high‐starch diets (Jansson and Lindberg 2012) and slower glycogen restorations in low‐starch diets (Lacombe et al. 2004; Valberg et al. 2023). In addition, Valberg et al. (2023) found that this delay in glycogen restorations was mirrored by a delay in the appearance of increased GLUT3, GLUT6, and GLUT10 expression. There is also a study on Standardbred horses with rhabdomyolysis that shows that those horses have alterations in expressions of genes and proteins involved in calcium ion regulation, cellular/oxidative stress, inflammation, and regeneration as characteristic of gluteal muscle (Valberg et al. 2022).

In the present study, the metabolic parameters differed between the different starch intake groups, indicating that energy substrates available during exercise were different in the different groups. The main difference was higher acetate values in the horses on low‐ or no‐starch intakes, which is in accordance with earlier studies on forage‐only diets (Jansson and Lindberg 2012; Connysson et al. 2017). Acetate, produced during the fermentation of fibres in the hindgut, is taken up by most cells in the body, converted by acetyl‐CoA synthetases to acetyl‐CoA, and metabolized via the citric acid cycle. The muscle cells' selection for fuels during exercise depends on exercise intensity and the availability of different energy substrates (Hodgson et al. 2014). Both Jansson and Lindberg (2012) and Martin et al. (2023) have shown that no‐starch diets alter metabolic response in trotters during exercise, probably due to more available acetate and less energy contribution from glycolysis. In addition, no‐starch diets have been shown to change energy metabolism during exercise and give a slower response to lactate accumulation (Karlsson et al. 2002; Jansson and Lindberg 2012).

In the present study, there is no clear connection between starch intake and plasma insulin concentration since there is no difference between medium‐ and low‐starch intake groups. However, the high‐starch intake group had higher insulin concentrations than the group with low starch intakes. This lack of difference is probably because the horses were fed at different time points before blood sampling, i.e., horses were fed in the morning, and the blood samples were collected approximately 0.5–4 h after feeding in connection with exercise. Plasma insulin concentrations are most elevated during the first hours after feeding (Duren et al. 1999; Pagan and Harris 1999; Jansson et al. 2006). Higher insulin concentration levels in horses fed starch than in horses fed no‐/low‐starch diets have been shown earlier (Williams et al. 2001; Connysson et al. 2010; Jansson and Lindberg 2012; Jensen et al. 2016). In Jansson and Lindberg (2012), starch‐rich diets resulted in elevated insulin levels in Standardbred trotters during warm‐up and recovery in connection with submaximal exercise.

In the present study, there was no effect of starch intake on plasma glucose concentrations. This can also be due to the blood sampling timing in relation to feeding since plasma glucose concentrations are affected by differences in the timing of feeding in relation to exercise (Duren et al. 1999; Pagan and Harris 1999). Elevated plasma glucose concentrations after exercise are due to an exercise‐induced increase in cortisol and glucagon levels that stimulates substrate mobilization and thereby glucose release from the liver (Hodgson et al. 2014).

Fillies had lower starch allowances than colts and geldings and younger horses had higher allowances than older horses. The lower starch allowance in fillies might be due to them being smaller (Saastamoinen 1990; Persson et al. 1996). That fillies had higher plasma muscle enzyme activity than colts and geldings is in accordance with earlier studies on two‐ and 3‐year‐old Thoroughbred racehorses (Harris et al. 1990; Harris et al. 1998). In the present study, a higher muscle enzyme activity in fillies was observed even though mean starch intake in the fillies was lower. In addition, fillies have an increased risk of developing exertional rhabdomyolysis (Harris 1991; McGowan et al. 2002). In humans, the sex difference is the opposite, with higher CK levels in males than females after exercise (Morawetz et al. 2020). Oestrogen has been suggested to be an important factor in this sex difference and that might be explained by oestrogen's membrane‐stabilizing effect (Morawetz et al. 2020). However, in horses, there appears to be no correlation between stages of the oestrus cycle and episodes of recurrent exertional rhabdomyolysis (Frauenfelder et al. 1986).

Intense exercise induces different levels of muscle damage. This damage can cause myofibrillar disruption, swelling, efflux of myocellular enzymes and myoglobin, as well as an inflammation process in the muscle that leads to adaptive remodelling of muscle (Peake et al. 2017). There are, however, no common well‐defined definitions for measuring degrees of exercise‐induced muscular damage. In athletes, such as racehorses, where recovery between exercise sessions is short, it might be of interest to decrease muscle damage. This study indicates that one strategy for doing this could be to alter energy metabolism with diets low in starch. One way of formulating low starch diets is to increase the forage proportion which requires high energy content of the forage. If the voluntary intake of forages is a limitation, forage offered as pellets or cubes may increase feed intake compared to when fed as hay (Cymbaluk and Christensen 1986). In addition, voluntary forage intake in trained Standardbred trotters has been shown to increase when kept outdoors in groups compared to when kept in individual box housing (Connysson et al. 2019).

In conclusion, this study showed higher plasma muscle enzyme activity of AST and some elevations in CK activity in horses fed high‐starch allowances compared to horses fed low allowances or no starch. In addition, muscle enzyme activities increased in response to the duration of high‐intensity exercise. Management systems aiming for low levels of plasma muscle enzyme activity could accordingly offer diets with low starch contents (< 450 g/day) and perhaps training regimes with shorter durations of high‐intensity exercise. Low starch intake might be of extra relevance for fillies. However, the mechanisms behind and the elevations' biological importance, need further investigation.

## Ethics Statement

The authors confirm that the ethical policies of the journal, as noted on the journal's author guidelines page, have been adhered to and the appropriate ethical review committee approval has been received. The Umeå Regional Ethics Committee approved the study, which was performed in compliance with European Union directives on animal experiments (2010/63/EU; European Union 2010), and laws (Swedish Constitution, 1988:534) and regulations (Swedish Board of Agriculture Constitution, 2012:26) governing experiments on live animals in Sweden.

## Supporting information

Supplementary Material Composition of concentrates 241118.

## Data Availability

The data that support the findings of this study are available from the corresponding author upon reasonable request.
