# Supplementary material for: Starch Allowance and Muscle Enzyme Activity in Healthy Standardbred Trotters Trained by Professional Trainers
Source: J Anim Physiol Anim Nutr (Berl). 2025 May 6;109(5):1130–7. doi: 10.1111/jpn.14127 (PMC12451409; doi:10.1111/jpn.14127)
Supplement: Supplementary file 1 — Supplementary Material Composition of concentrates 241118. [file JPN-109-1130-s001.docx]

**Supplementary Material Composition of concentrates.**

Starch allowance and muscle enzyme activity in healthy Standardbred trotters trained by professional trainers

M. Connysson and A. Jansson

a Department of Animal Biosciences, Swedish University of Agricultural Sciences, Box 7023, 750 07 Uppsala, Sweden

Corresponding author: Malin Connysson. Email: malin.connysson@slu.se

The composition of the concentrates used by trainer one was (concentrate A: Beet pulp, Oats, Wheat, Oats, Molasses, Wheat bran, Green meal, Alfalfa, Oat bran, Potato protein, Rapeseed oil, Sodium chloride, starch content 256 g/kg DM and concentrate B Oat bran, Wheat bran, Oats, Alfalfa, Oats, Wheat, Green meal, Beet pulp, Molasses, Potato protein, Sodium chloride, Rapeseed oil, starch content 171 g/kg DM).

The composition of the concentrates used by trainer two was (concentrate C: Beet pulp, Oats, Wheat, Oats, Molasses, Wheat bran, Green meal, Alfalfa, Oat bran, Potato protein, Rapeseed oil, Sodium chloride, starch content 252 g/kg DM and concentrate D: Oat bran, Wheat bran, Oats, Alfalfa, Oats, Wheat, Green meal, Beet pulp, Molasses, Potato protein, Sodium chloride, Rapeseed oil, starch content 169 g/kg DM).

The composition of the concentrates used by trainer three was (concentrate E: Oat Flakes, Maize Flakes, Barley Flakes, Sugar Cane Molasses, Soya Bean Meal, Soya Bean Flakes, Soya Bean Hulls, Soya Bean Extruded, Pea Flakes, Soya Oil , Wheat, Sunflower Seed Meal, Wheatfeed, Barley, Calcium Carbonate, Mono-dicalcium Phosphate, Sodium Chloride, Magnesium Oxide, starch content 340 g/kg DM and concentrate F: Oats, Barley, Corn, Extruded soybeans, Alfalfa meal, Soybean hulls, Sugarcane molasses, Wheat, Sunflower seed meal, Soybean meal, Beet pulp, Monocalcium and dicalcium phosphate, Calcium carbonate, Sodium chloride, Magnesium oxide. starch content 306 g/kg DM and Concentrate G:Oats, starch content 403 g/kg DM Concentrate H: Groun Soybean Hulls, Dried Plain Beet Pulp, Ground Oats, Ground Extruded Whole Soybean, Ground Barley, Dehydrated Alfalfa Meal, Cane Molasses, Dehulled Sunflower Meal, Wheat Shorts, Soybean Oil, Mono-dicalcium Phosphate, Salt, Magnesium Oxide, Calcium Carbonate, Yeast Primary Dried, Dried Chicory Root, Ferrous Sulfate, Active Dry Yeast, Biotin, starch content 153 g/kg DM).

The composition of the concentrates used by trainer four was (Concentrate I:Barley, Wheat, Corn, Molasses, Green meal, Sugar beet pulp, Soybean, Rapeseed oil, Soybean oil, Sodium chloride, starch content 345 g/kg DM, Concentrate J: Oats, starch content 467 g/kg DM).

Trainer five did not feed any concentrates.
